# Comparative analysis of tissue-specific transcriptomes in the funnel-web spider *Macrothele calpeiana* (Araneae, Hexathelidae)

**DOI:** 10.7717/peerj.1064

**Published:** 2015-06-30

**Authors:** Cristina Frías-López, Francisca C. Almeida, Sara Guirao-Rico, Joel Vizueta, Alejandro Sánchez-Gracia, Miquel A. Arnedo, Julio Rozas

**Affiliations:** 1 Departament de Genètica and Institut de Recerca de la Biodiversitat (IRBio), Universitat de Barcelona, Barcelona, Spain; 2 Departament de Biologia Animal and Institut de Recerca de la Biodiversitat (IRBio), Universitat de Barcelona, Barcelona, Spain

**Keywords:** *De novo* transcriptome assembly, Molecular markers, Chemosensory system, RNA-seq, Mygalomorphae Phylogeny

## Abstract

The funnel-web spider *Macrothele calpeiana* is a charismatic Mygalomorph with a great interest in basic, applied and translational research. Nevertheless, current scarcity of genomic and transcriptomic data of this species clearly limits the research in this non-model organism. To overcome this limitation, we launched the first tissue-specific enriched RNA-seq analysis in this species using a subtractive hybridization approach, with two main objectives, to characterize the specific transcriptome of the putative chemosensory appendages (palps and first pair of legs), and to provide a new set of DNA markers for further phylogenetic studies. We have characterized the set of transcripts specifically expressed in putative chemosensory tissues of this species, much of them showing features shared by chemosensory system genes. Among specific candidates, we have identified some members of the iGluR and NPC2 families. Moreover, we have demonstrated the utility of these newly generated data as molecular markers by inferring the phylogenetic position *M. calpeina* in the phylogenetic tree of Mygalomorphs. Our results provide novel resources for researchers interested in spider molecular biology and systematics, which can help to expand our knowledge on the evolutionary processes underlying fundamental biological questions, as species invasion or biodiversity origin and maintenance.

## Introduction

The funnel-web spider *Macrothele calpeiana* (family *Hexathelidae*) is a charismatic component of the European arthropod fauna. It belongs to the spider infraorder Mygalomorphae, which includes about 3,000 species of, among others, trap-door spiders, funnel-web spiders, and tarantulas (Platnick, 2006). *M. calpeiana* is a hairy, large spider that constructs extended and conspicuous funnel-web sheets close to the ground, and it is the only spider protected under European legislation (Collins & Wells, 1987). This spider is endemic to the southern Iberian Peninsula and was initially considered to be particularly vulnerable due to its close association with the highly threatened cork-oak forests found in the region (Collins & Wells, 1987). Subsequent studies, however, demonstrated that the species has a much wider distribution and could be frequently found in highly disturbed areas. In the last years, *M. calpeiana* has been introduced in European countries outside its natural range, probably associated with the commercial export of Spanish olive trees, raising some concerns about their possible impact on the invaded ecosystems (Jiménez-Valverde, Decae & Arnedo, 2011).

*M. calpeiana* is also an organism of particular interest in biogeographic studies. The *Macrothele* genus shows a highly disjointed distribution, with the bulk of its diversity in South-East Asia (21 species), a few species inhabiting tropical Africa (4 species) and only two known species in Europe, *M. calpeiana* itself and *M. cretica*, a Cretan endemic spider that is also of conservation concern. A recent phylogenetic study (Opatova & Arnedo, 2014) has revealed that the two European *Macrothele* species are not sister taxa, and that they most likely colonized independently Europe from Asia. Another interest in the genus relates to the venom toxins of some *Macrothele* spiders, which can be strong enough to cause envenomation, as in the case of some large Taiwanese *Macrothele* spiders (Hung & Wang, 2004). In fact, studies on the molecular structure and chemical properties of venom toxins (Zeng, Xiao & Liang, 2003; Corzo et al., 2003; Satake et al., 2004; Yamaji et al., 2009) have established the utility of *Macrothele* venom as cell growth inhibitors in cancer research (Gao et al., 2005; Liu et al., 2012).

The scarcity of genomic and transcriptomic data in chelicerates, which just cover a few species (Grbić et al., 2011; Mattila et al., 2012; Cao et al., 2013; Clarke et al., 2014; Sanggaard et al., 2014; Posnien et al., 2014) and the lack of tissue-specific transcript data in mygalomorphs, clearly limit the research on the molecular determinants of fundamental biological processes in this group of species. Within this context, with the aim of shedding light on the composition of Mygalomorph transcriptomes, we conducted the first RNA-seq study in one species of this group, *M. calpeiana*, including several tissues, and using a 454GS-FLX-based technology (Prosdocimi et al., 2011). The new sequence data will be an important, initial contribution to further basic, applied, and translational research in this non-model organism. Here we address two specific objectives: (i) to identify possible candidate chemosensory transcripts for future studies, and (ii) to provide new markers for further phylogenetic and evolutionary genomic-based studies in this group. As an example, we used some of the new generated transcripts to clarify the phylogenetic position of *M. calpeiana* in the Mygalomorph phylogeny.

The chemosensory system plays a key role in fundamental vital processes, including the localization of food, hosts, or predators and social communication; nevertheless, there are very few studies focused in non-insect species results (Vieira & Rozas, 2011; Montagné et al., 2015), and almost unknown in mygalomorphs. In insects, the main molecular components of the chemosensory system are encoded by two main groups of gene families (Sánchez-Gracia, Vieira & Rozas, 2009; Vieira & Rozas, 2011; Almeida et al., 2014) the chemoreceptors and the secreted ligand-binding proteins. The first include the gustatory (GR), olfactory (OR), and ionotropic (IR) receptors, while the second group, known as ligand-binding families, are the odorant-binding protein (OBP), chemosensory protein (CSP), chemosensory type A and B (CheA/B), and probably some members of the Niemann-Pick disease type C2-related (NPC2) family (Pelosi et al., 2014). The preliminary analyses of the genomic sequences of the chelicerates *I. scapularis* (M Gulia-Nuss et al., 2015, unpublished data), *Stegodyphus mimosarum, Acanthoscurria geniculata*, (Sanggaard et al., 2014), *Mesobuthus martensii* (Cao et al., 2013), and *Tetranychus urticae* (Grbić et al., 2011), as well as in other arthropods, like the centipede *Strigamia maritima* (Chipman et al., 2014), revealed the absence of the typical insect OR and OBP gene families in these species.

Several experimental studies of chelicerates have identified the presence of specialised chemosensory hairs predominantly in the distal segment of the first pair of legs and in palps (Foelix, 1970; Foelix & Chu-Wang, 1973; Kronestedt, 1979; Cerveira & Jackson, 2012). In order to investigate the presence of transcripts related to the chemosensory system in spiders, we sequenced the specific transcriptomes of these two structures in *M. calpeiana*. To enrich our samples in tissue-specific transcripts, we built subtractive normalized cDNA libraries for each of these tissues separately. Additionally, for comparative purposes, we also analysed the ovary RNA-seq data. In this way, this study represents a starting-point to characterize the gene expression in the putative chelicerate chemosensory system structures.

Because of their low vagility and restricted distributions, mygalomorph spiders are well-suited for monitoring the ecological and evolutionary conservation status of terrestrial ecosystems (Bond et al., 2006), while at the same time are also highly threatened by habitat destruction (Harvey, 2002). To date, however, the lack of informative nuclear markers has limited research on these organisms and has hampered the assessment of their conservation or invasive species status. The method we employed here provides useful data for developing nuclear molecular markers to be used in other evolutionary genomic, phylogenetic, and phylogeographic studies of *Mygalomorphae*.

## Methods

### Sample collection and preparation

Four adult females of the spider *Macrothele calpeiana* were collected (Junta de Andalucía, Spain; permission: SGYB-AFR-CMM) in two different localities in the southern Iberian Peninsula, namely Iznalloz (Granada, N37.36468 W3.47183, 1,011 m) (individuals MAC-GR1, MAC-GR2, MAC-GR3) and Finca de los Helechales, rd. Cabeza la Vaca (Huelva, N38.09032 W6.46621, 749 m) (individual CRBAMM000991). For each individual, palps, distal segments of the first pair of legs (denoted as legs), ovaries, brains and muscle tissues (from the rest of legs) were dissected and stabilized in RNA later (Applied Biosystems/Ambion).

### Total RNA extraction and cDNA preparation

Each tissue was disrupted and homogenized separately using a rotor-stator homogenizer. Total RNA was extracted with the RNeasy midi kit (Qiagen, Hilden, Germany). For all dissected tissues, except the ovary, the protocol included a proteinase K digestion step in order to digest contaminant proteins. All samples were enriched in poly(A) mRNA prior to library preparation using the Oligotex RNA midi kit (Qiagen, Hilden, Germany).

The purified mRNA was used as a template for synthesizing the first cDNA strand using the SMARter PCR cDNA Synthesis Kit (Clontech, Mountain View, California, USA). In this protocol, a poly(A)-specific primer initiates the first strand synthesis of cDNA, thus selecting for polyadenylated RNA while simultaneously keeping the concentration of ribosomal RNA low. The resulting single stranded cDNA was amplified with the Advantage2 PCR kit (Clontech, Mountain View, California, USA), using 23 (brain, leg and muscle) and 20 (palp and ovary) amplification cycles. Double stranded cDNA was purified using CHROMA SPIN-1000 columns (Clontech, Mountain View, California, USA) and subsequently cleaved with *Rsa1* to generate shorter, blunt-ended cDNA fragments, which are necessary for adaptor ligation and subtraction. The digested cDNA were then purified using a standard phenol:chloroform:isoamyl extraction.

### Subtractive hybridization and RNA sequencing

Transcripts expressed specifically in the palps, legs, and ovaries were enriched using the PCR-Select cDNA Subtraction Kit (Clontech, Mountain View, California, USA). This technique is based on a method of selective amplification of differently expressed sequences. We used leg, palp, and ovary cDNA as tester (samples of interest) and brain and muscle cDNAs samples as driver (transcripts exclusively for subtraction purposes) samples. According to the kit’s protocol, the tester samples are subdivided into two aliquots that receive different adaptors. These aliquots are mixed to driver cDNA (in a higher concentration), denatured, and allowed to reanneal to form double chain cDNA. The process in repeated once, but with the two aliquots of tester cDNA mixed together and some more tester cDNA added. Then a PCR is done in a way that only double chain cDNA formed by fragments with different adaptors at each end will be amplified (i.e., cDNA formed by the hybridization of single chain cDNA from different tester aliquots). In this way, the sample is enriched with cDNA specific to the tester tissue since the tester cDNA that hybridizes with driver cDNA does not get amplified. The subtraction process also normalizes the library so that the frequencies of each unique cDNA became less unequal, increasing the chances of sequencing a large number of unique cDNAs. The subtracted cDNA products were treated with RNase (Qiagen, Hilden, Germany) and purified with QIAquick PCR Purification Kit (Qiagen, Hilden, Germany).

Two micrograms of subtracted cDNA from each tester tissue was prepared for sequencing on a 454/ Roche GS-FLX Titanium sequencer using three different MID tags, one for each tissue. Double-stranded cDNA was nebulized to generate 500-kb fragments and a shotgun library prepared for GS-FLX sequencing as per the manufacturer’s instructions (Roche, Basel, Switzerland), which was run on a 1/4 picotitre plate region.

### Read processing, handling, and *de novo* transcriptome assembly

We used *sffinfo* script (Roche’s Newbler package; 454 SFF Tools) to extract the DNA sequences (FASTA format) and quality scores (FastQ format) independently for each MID tag from the SFF file. We removed adapters and putative contaminant sequences (upon the UniVec database and the *E. coli* genome sequence data) with SeqClean script (http://compbio.dfci.harvard.edu/tgi/software/), with parameters: -v <sequence of adapters> -c 8 -l 40 -x 95 -y 11 -M -L -s <database of contaminant sequences>. We trimmed low-quality bases at the ends of the reads and removed those shorter than 100bp or with a mean quality score (Q) below 20 using the NGS QC Toolkit (Patel & Jain, 2012).

First, we conducted a complete *de novo* assembly using all reads from the three tissues altogether in Newbler v2.6 GS (454 life Sciences, Roche Diagnostics) with parameters -urt -cDNA -Denovo -mol 100 -moi 95 -url. Subsequently, we used the contigs and the non-assembled reads (i.e., singletons) from this first step as input for a second assembly round in CAP3 (Huang, 1999), with parameters –o 60 –p 95. Redundant transcripts and putative isoforms were removed using cd-hit-est program, to generate a list of unique transcripts (Fu et al., 2012). We then used the gsMapper program (included in Newbler package) to map original (after filtering) reads (from the 3 tissues) to the unique transcripts, discarding all reads exhibiting hard clipping (more than 10% of read length) with an in-house Perl script.

### Functional annotation

We carried out most of the functional annotation of the assembled transcripts with blast (v. 2.2.29) (Altschul, 1997; Camacho et al., 2009), Blast2GO (Conesa et al., 2005), InterProScan (Jones et al., 2014) and TRUFA (Kornobis et al., 2015). We first conducted a series of similarity-based searches with blastx (E-value cut-off 10^−3^) against the NCBI non-redundant (NCBI-nr) database, retrieving the 5 hits with the lowest E-value for each query transcript. We then used Blast2GO and TRUFA to: (i) assign the Gene Ontology (GO) terms to each of these transcripts and determine the involved KEGG pathways (Kanehisa & Goto, 2000), (ii) identify particular protein domain structures in the sequenced transcripts using the InterProScan search engine, and (iii) determine which GO terms, InterPro domains, and KEGG pathways were significantly enriched in particular tissues by applying the Fisher’s exact test and controlling by the False Discovery Rate (FDR) (Benjamini & Hochberg, 1995).

To determine the efficiency of the subtractive approach employed here to enrich samples with tissue specific transcripts, we estimated the fraction of assembled transcripts encoding for putative housekeeping (HK) genes (i.e., transcripts expected to be expressed across different tissues). For the analysis, we considered that a *M. calpeiana* transcript encodes a HK gene if we obtained a significant blastx hit (E-value cut-off 10^−3^) against a database that includes all HK genes shared between humans (data set from Eisenberg & Levanon, 2013) and *Drosophila melanogaster* (data set from Lam et al., 2012) (which correspond to the 80% and 94% of the human and *Drosophila* HK genes, respectively; Table S1A). Furthermore, we also estimated the number of transcripts that encode genes included in the CEG (Cluster of Essential Genes) database (a set of 458 Eukaryotic Orthologous Groups proteins identified by the Core Eukaryotic Genes Mapping Approach, CEGMA) (Parra, Bradnam & Korf, 2007; Parra et al., 2009). CEG proteins are highly conserved and present in a wide range of eukaryotic organisms, being therefore a good dataset to assess the reliability of our RNA sequencing and transcript annotation. *VennDiagram* R package was used to obtain all graphic representations of the logical relations (http://cran.r-project.org/web/packages/VennDiagram/index.html).

In order to identify putative *M. calpeiana* chemosensory related transcripts, we carried out an additional specific and customized search. We first built a protein database (CheDB) with vertebrate and insect sequences that match against the InterPro protein family signatures associated with chemosensory function (Table S1B). Then, we conducted a blastx search (E-value of 10^−3^) using the assembled contigs as query against the CheDB database. To minimize the percentage of false positive results, we checked whether the candidate chemosensory transcripts from the blast searches truly encoded the Pfam HMM core profiles corresponding to chemosensory protein domains, using the programs HMMER (Eddy, 2009) (E-value of 10^−3^) and InterProScan. Only *M. calpeiana* transcripts with positive hits in this second search step were unequivocally annotated as putative chemosensory genes. Finally, we also ran an additional tblastn search (E-value of 10^−3^) of a set of proteins annotated as chemosensory in currently available chelicerate genomes—the common house spider *Parasteatoda tepidariorum* (https://www.hgsc.bcm.edu/arthropods/common-house-spider-genome-project), the social spider *Stegodyphus mimosarum* (Sanggaard et al., 2014), the mygalomorph spider *Acanthoscurria geniculate* (Sanggaard et al., 2014), the scorpion *Mesobuthus martensii* (Cao et al., 2013), and the tick *Ixodes scapularis* (https://www.vectorbase.org/) against *M. calpeiana* transcripts. In this last search, we also included as queries the translated sequences of the transcripts already identified as candidate *M. calpeiana* chemosensory genes in the first searches. In order to exclude spurious homologs caused by short-length false-positive hits, we only considered for further analyses those transcripts whose blast alignments span either at least 2/3 of the total number of amino acids of the query proteins or those covering at least 80% of the transcript length.

### Phylogenetic analysis

To determine the utility of the newly sequenced transcripts as markers for molecular phylogenetics, we applied them to study the phylogenetic position of *M. calpeiana* in the tree of Mygalomorphs, a currently unresolved question. As a starting point, we used the phylogenetic analysis reported in Bond et al. (2014). In particular, we first retrieved the amino acid data of all 16 mygalomorph and 3 non-mygalomorph outgroup species (*Stegodyphus*, *Hypochilus* and *Liphistius*) from the matrix d327 (44 taxa; 327 genes; 110,808 amino acid positions). Then, we searched for putative homologs of these 327 genes in *M. calpeiana* transcripts using the blastp program. For this analysis, we obtained the conceptual translation of the transcript sequences (in all six frames) using TransDecoder (version r20140704) as implemented in the Trinity software (Haas et al., 2013). We selected all *Macrothele* translated amino acid sequences that produced a positive blast hit with an E-value < 10^−15^ and with local alignment length >80 amino acids (i.e., in order to maximize the probability of using 1:1 orthologues). Then, we aligned each of these selected translated sequences of *M. calpeiana* with their corresponding homologs in the 19 chosen species (a single multiple sequence alignment, MSA, per gene) using MAFFT (option–merge) (Katoh & Standley, 2013). Finally, we concatenated all individual MSA with amino acid data in at least 50% of the species.

We also built family specific MSA with amino acid sequences of NMDA-ionotropic glutamate receptors (NMDA-iGluR) and with members of the Niemann-Pick C disease 2 (NPC2) family, to investigate the phylogenetic relationships between the candidate *M. calpeiana* transcripts and some representatives of these two families in arthropods. We included in these MSA the proteins already annotated in *D. melanogaster* (hexapod), *S. maritima* (myriapod) and *I. scapularis* (chelicerate), as well as the NPC2 genes expressed in *Apis melifera* and *Camponotus japonicus* antenna (Pelosi et al., 2014). For iGluR (including IR8a/IR25a proteins) we prepared two different MSA, one for each functional domain. We used HMMER and the Pfam profiles of these two domains (PF01094 “ANF_receptor,” and PF00060 “Lig_chan”) to identify and trim separately the extracellular amino-terminal and the ligand-gated ion channel domains, which were used to build two separate MSA (and separate trees) with HMMERALIGN.

We conducted all phylogenetic reconstructions by maximum likelihood (ML) using the PROTGAMMAWAG model in the program RAxML version 8 (Stamatakis, 2014). We carried out a multiple non-parametric bootstrap analysis (500 bootstrap runs) to obtain node support values.

## Results and Discussion

### RNA-seq of *Macrothele calpeiana*

We sequenced a total of 164,111 raw reads across the three tester samples (i.e., leg, palp, and ovary), with a N50 value of 409bp (Table 1). After trimming, cleaning and removing very short reads (less than 100bp), we obtained a final set of 128,816 reads, which was used for further analyses. Our two-step *de novo* assembly strategy (applying Newbler v 2.6, and subsequently CAP3) yielded a total of 3,705 contigs (N50 of 647bp), composed by more than one read, plus 3,560 singletons. After running the cd-hit-est and gsMapper software these contigs clustered into 6,696 unique sequences (i.e., putative *M. calpeiana* individual coding genes), of which 3,467 corresponds to contigs assembled by more than one read (i.e., excluding singletons) (Table 1; Table S2). Table 2 and Table S3 show the distribution of these 6,696 (and also the 3,467) unique sequences across tissues. *M. calpeiana* reads data are available at the Sequence Read Archive (SRA) database under the accession numbers SRA: SRS951615, SRA: SRS951616 and SRA: SRS951618 (Bioproject number: PRJNA285862).

**Table 1:** Summary of RNA-seq data and assembly.

Raw number of reads	164,111
N50	409
Reads used in the Newbler assembly^a^	128,818
Assembled reads	122,183
Isotigs (number of singletons)	3,635 (6,614)
N50 (Isotigs)	601
CAP3 assembly	
Contigs (number of singletons >100nuc)	3,705 (3,560)
N50 (Contigs)	647
Unique sequences^b^	
Total number of sequences (transcripts)	6,696
N50	455
Coverage	14.33X
Reads mapped	95,250
Sequences (excluding singletons)	3,467
N50	613
Coverage	22.94X
Reads mapped	90,267

**Notes.**

a Number of reads after trimming, cleaning and excluding short reads.

b Number of reads after clustering and mapping filtering.

**Table 2:** Summary of RNA-seq data and assembly per tissue.

	Leg	Palp	Ovary	Total
Driver^a^	Muscle	Muscle	Brain	
Raw number of reads	59,232	54,321	50,558	164,111
N50	404	405	419	409
Reads used in assembly^b^	46,474	41,545	40,799	128,818
N50	362	364	378	368
Unique sequences (transcripts)^c^	2,705	3,798	1,796	6,696
Longest transcript (in nucleotides)	3,053	3,057	4,116	4,116
HK, housekeeping sequences	426	638	328	1,005
CEG sequences	385	547	236	789
Sequences excluding HK-CEG genes	2,139	2,952	1,369	5,390
Sequences with GO annotation	1,147	1,612	816	2,619
Sequences within Interpro	1,464	1,966	988	3,353
Sequences within KEGG	389	509	173	776
Sequences with functional annotation^d^	1,704	2,363	1,152	3,970
Sequences with annotation^e^	2,060	2,915	1,428	4,978

**Notes.**

a Driver of subtractive cDNA library.

b Number of reads after trimming, cleaning and excluding short reads.

c Considering the total (*n* = 6, 696) data set.

d GO, Interpro or KEGG hits.

e GO, Interpro, KEGG or blast hits.

### RNA-seq quality and functional annotation

**Figure 1:**
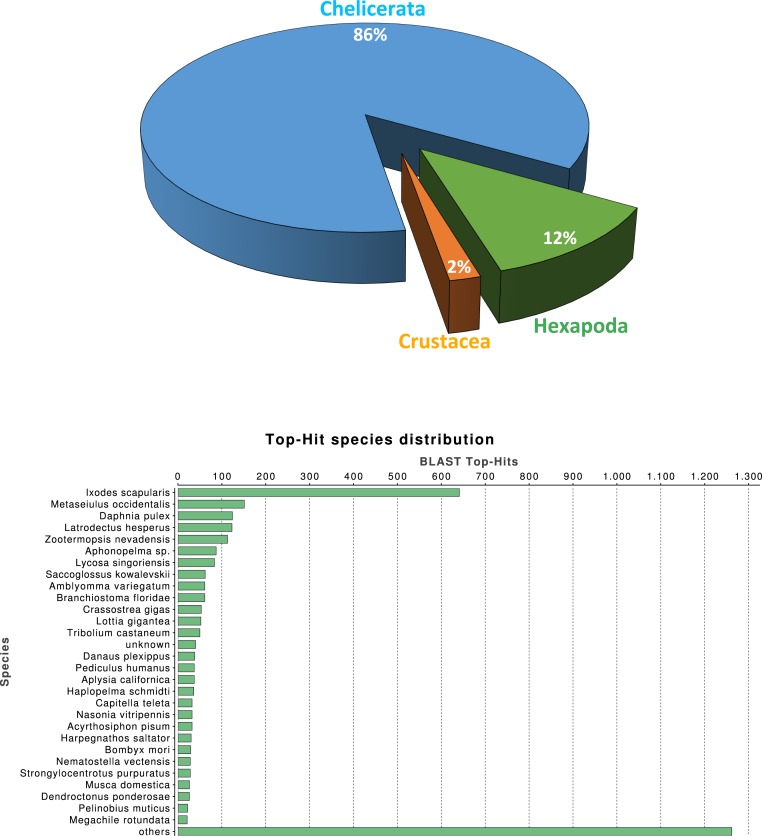
Macrothele taxonomic distribution. Taxonomic distribution of the 6,696 transcripts with significant blast hits against the NCBI-nr data base (using the top-hit; cut-off E-value of 10^−3^) by means of the Blast2GO package (4,399 transcripts with blast hit). (A) Distribution of the top-hits across arthropod groups (29.4% of the transcripts with blast hit). (B) Top-hit species distribution.

We investigated the quality of our tissue specific transcriptome by a series of similarity-based searches of our transcripts against sequences in the NCBI-nr database. As expected, the single largest category of top blast hits (blastx E-value cut-off 10^−3^), corresponding to 25.3% of top blast hits, was to chelicerate protein coding genes, followed by hits to other arthropod species (4.1%). Within the Arthropoda, hits within Hexapoda represents about 12% (Fig. 1A), while *Ixodes scapularis* is the species receiving the majority of hits (Fig. 1B).

**Figure 2:**
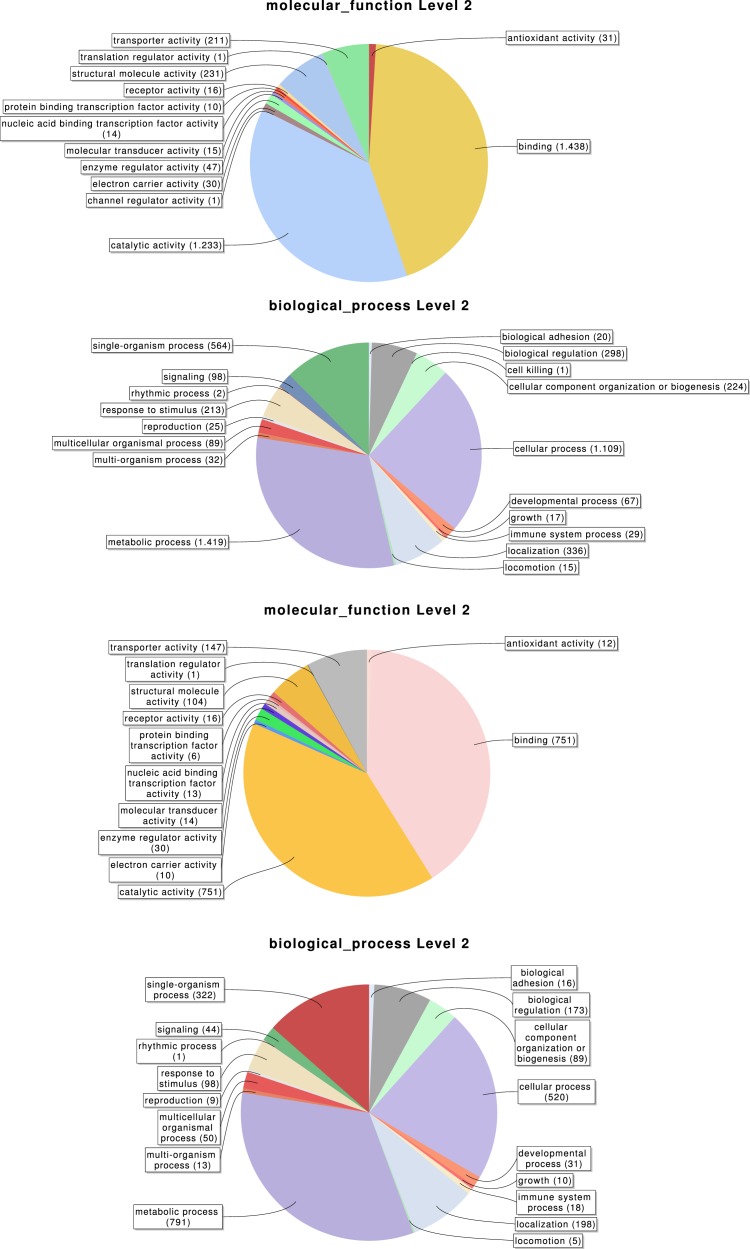
Distribution of the Gene Ontology (GO) terms associated with the complete set of *M. calpeiana* transcripts (2,619 transcripts with GO annotations over 6,696 sequences). (A) MF, molecular function. (B) BP, Biological process. Distribution GO terms excluding transcripts encoding HK or CEG genes (1,523 transcripts with GO annotations over 5,390 sequences). (C) MF, molecular function. (D) BP, Biological process.

Overall, 2,619, 3,353 and 776 out of the 6,696 identified transcripts have a GO, InterPro, or KEGG associated term, respectively (Table 2); in total 4,978 of them (74.3%) have some functional annotation information. We analysed the distribution of GO terms (at GO level 2) across the 2,619 *M. calpeiana* transcripts sequences with GO annotation. We found that the most frequent GO terms present in this sample are “metabolic” and “cellular processes” within the biological process domain (BP), and “binding” and “catalytic activities” within molecular function domain (MF). The distribution of GO terms in the complete data set (2,619 GO terms; Fig. 2) and in the data set excluding singleton sequences (1,734 GO terms; Fig. S1) is not significantly different (two tailed FET, *P*-value = 0.592 and 0.757 for BP and MF, respectively). Hence, we used the complete dataset for further functional annotation analyses.

### Tissue-specific expression

**Figure 3:**
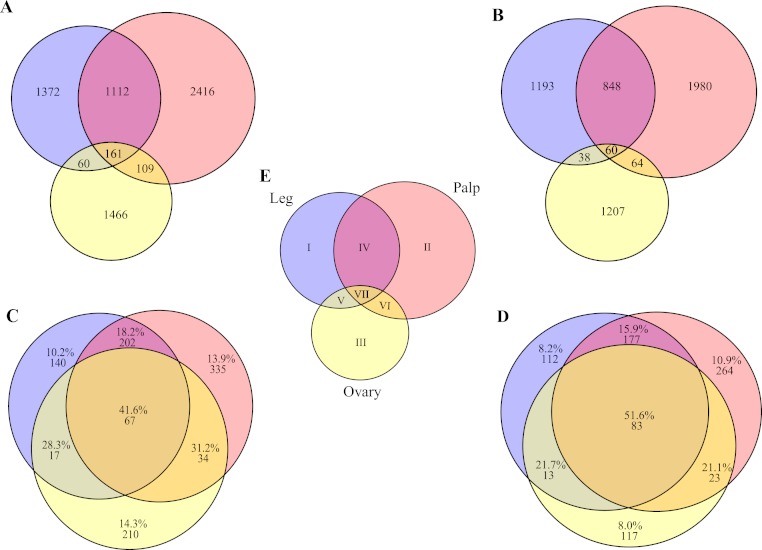
Transcript distribution across tissues. Venn diagrams showing the number of sequences expressed specifically in each tissue or in their intersections (blue, ochre and yellow indicate leg, palp and ovary, respectively). (A) All transcripts (*n* = 6,696). (B) Transcripts excluding putative housekeeping or CEG genes (*n* = 5,390). (C) Number and percentage of transcripts encoded by housekeeping genes (*n* = 1,005). (D) Number and percentage of transcripts with homologs included in the CEG database (*n* = 789). The area of each Venn diagram section is approximately proportional to the number of transcripts (A and B), or to the particular fraction value (C and D). (E) Roman numerals used to designate the different sections.

With our subtractive approach we aimed to enrich a number of tissue-specific transcripts. We detected 1,005 transcripts annotated as housekeeping genes (Table 2) and 789 transcripts with putative homology to 290 of 458 CEG members of the CEGs dataset. Out of the 789 transcripts with CEG homologs, 488 are also annotated as HK genes (Fig. S2 and Tables S3–S5). Despite the finding of about 15% of HK and CEG genes, the largest proportion of them are located at the intersection of the Venn diagram (Figs. 3C and 3D), indicating that tissue-specific transcripts should reliably represent tissue-specific functions. After excluding these likely ubiquitously expressed genes, the remaining sample (*n* = 5, 390 transcripts; 1,523 with GO annotation) exhibits the desired tissue-specific expression profile. In fact, the distributions of GO terms including (2,619 transcripts) or not (1,523 transcripts) HK/CEG genes are significantly different from each other (two tailed *P*-value < 0.018 for the most frequent GO categories within BP and MP) (Fig. 2).

To gain further insight into transcript function, we compared transcript expression across legs, palps, and ovaries (Fig. 3; Fig. S3). We found a high proportion of transcripts shared between leg and palp (1,112 and 848, including or not HK and CEG genes, respectively), and a few between these tissues and ovary (Figs. 3A and 3B). This result was expected given the ontogenetic similarities of legs and palps.

**Figure 4:**
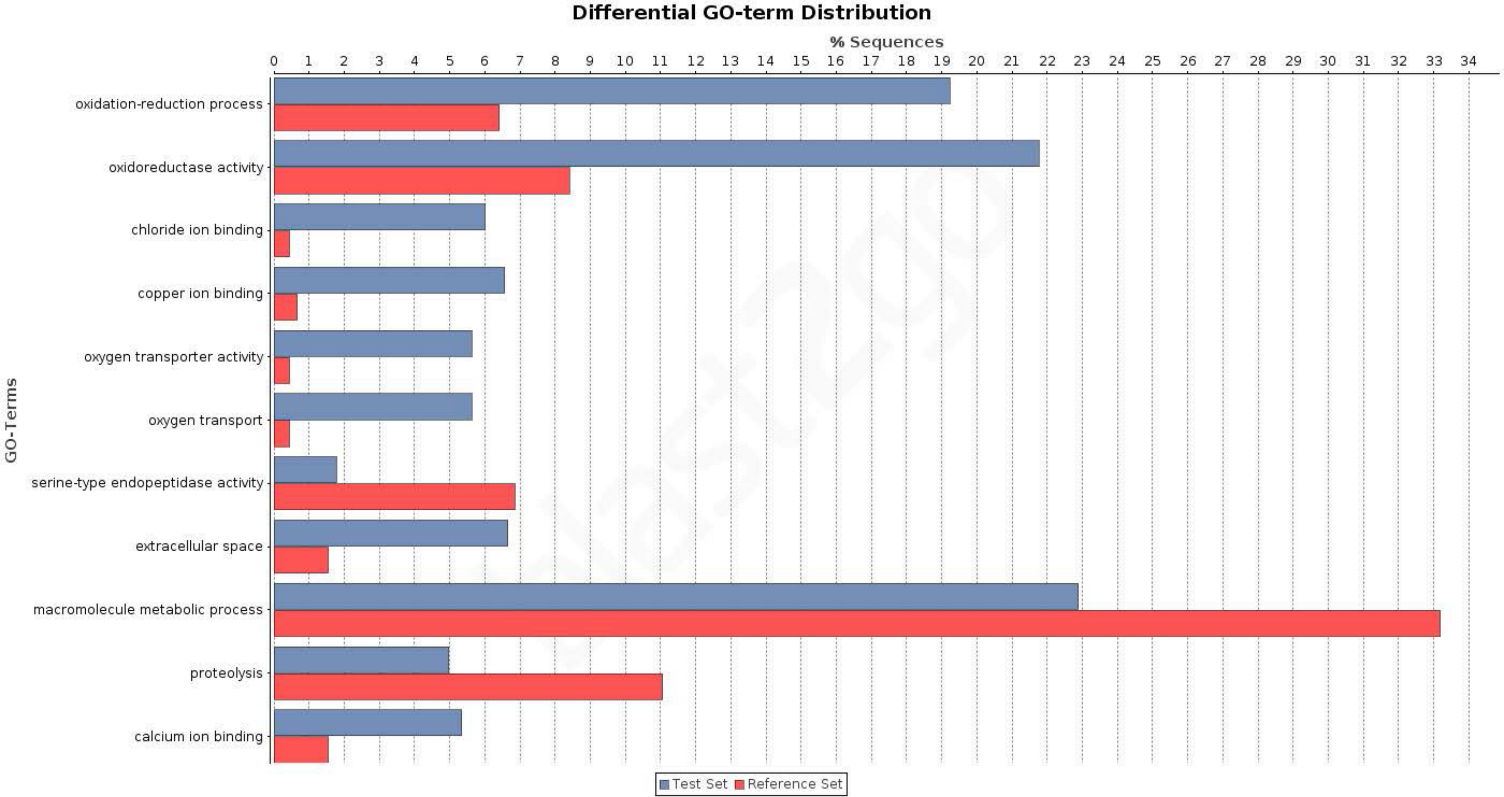
Differential distribution of GO terms across tissues. Differential distribution of the GO terms of the transcripts from leg and palp (Venn sections I, II and IV; in blue) and ovary (sections III, V, VI and VII; in red). Analysis conducted excluding HK and CEG encoding genes (1,523 transcripts over 5,390).

The overrepresentation analysis of the GO terms across the different Venn diagram sections (Table S3; see also Fig. 3E) detected 26 significant overrepresented GO terms in legs-palps (sections I, II and IV) or ovary transcripts (sections III, V, VI and VII) after the FDR (Fig. 4; Table S6A and Fig. S4). For instance, the GO terms “cation binding,” “metal ion binding,” and “oxidation–reduction process” are clearly overrepresented in legs-palps specific transcripts (*P*-value < 6.9 × 10^−8^). These significant differences are also found in comparisons involving only section III (i.e., considering only ovary-specific transcripts instead of all ovary-transcripts), or only section IV (considering only specific transcripts shared between leg and palp) (results not shown). Indeed, the major over- or underrepresentation effect appears in individual sections III and IV (Table S6).

To investigate the biological pathways that are differently expressed among the studied tissues, we analysed the distribution of transcripts associated with different KEGG terms (Tables S3 and S7). Again, we found significant differences between transcripts expressed exclusively in legs and/or palps (sections I, II, and IV) and the ovary-expressed transcripts (sections III, V, VI, and VII) (two tailed FET, *P*-value of 2.6 × 10^−3^). For instance, we detected 3 KEGG pathways (Tropane, piperidine and pyridine alkaloid biosynthesis; Tryptophan metabolism; and Tyrosine metabolism) specifically expressed in sections I, II and IV; none of the 11 detected transcripts of these three pathways had ovary expression (Table S7). Actually, these pathways are not directly related to chemosensory function. It has been shown that the golden orb web spider *Nephila antipodiana* (Walckenaer) coats its web with an alkaloid (2-pyrrolidinone), which apparently provides protection against ant invasion (Zhang et al., 2012). *Macrothele* large funnel-webs are equally exposed to predators, both insects and small vertebrates, and hence the use of a chemical defense against invaders would be highly advantageous. Further studies on the presence of these chemical clues on the funnel-webs are needed to confirm this hypothesis.

### Chemosensory-related genes

**Figure 5:**
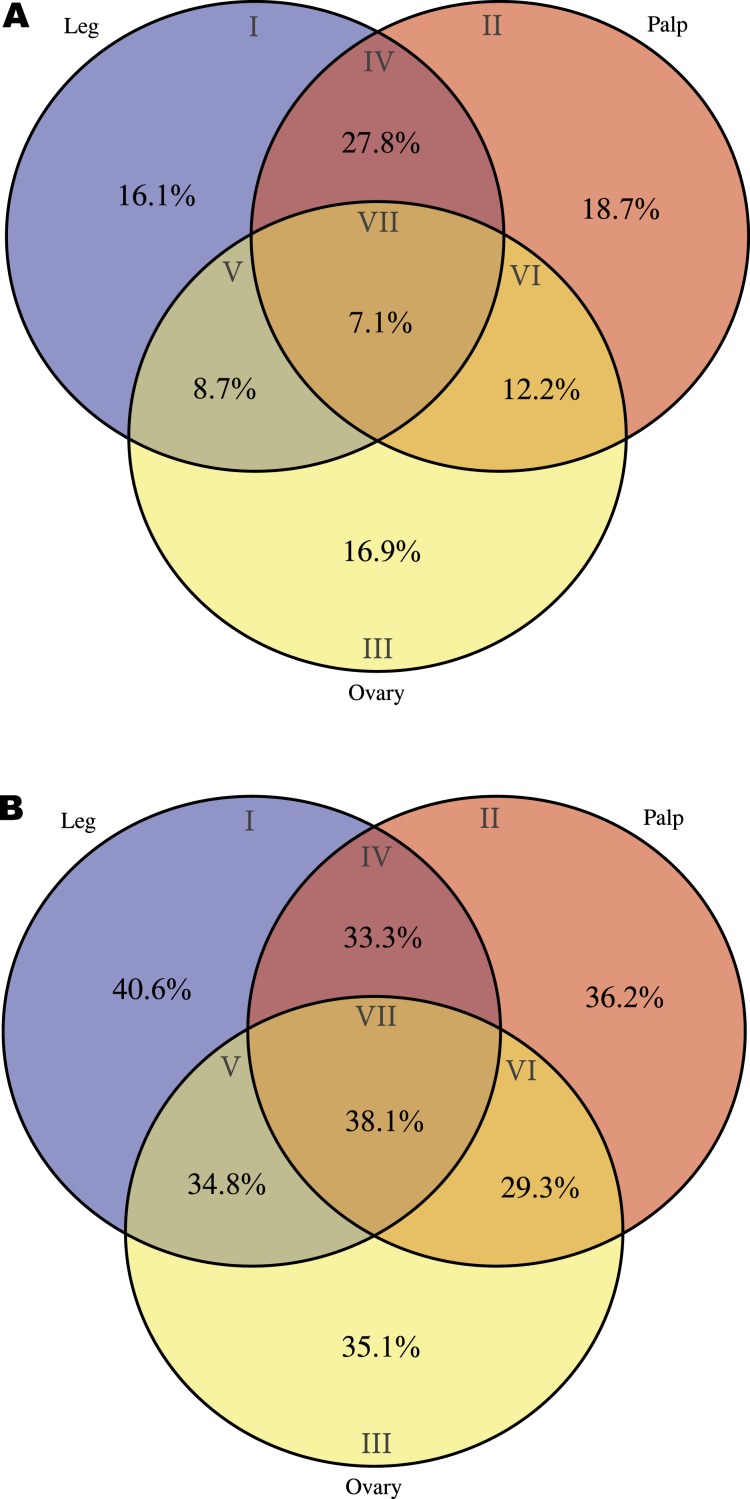
Distribution of specific interpro domains across tissues. Venn diagrams showing the percentage of specific interpro domains across tissues (the different Venn sections are indicated in roman numbers). Analysis conducted excluding HK and CEG encoding genes (2,364 transcripts with Interpro annotation over 5,390). (A) Signal peptide domain. (B) Transmembrane domain.

As a starting point for the identification of chemosensory organs in *M. calpeiana*, we studied two features commonly present in the chemosensory-related proteins, the existence of a signal peptide (characteristic of soluble binding proteins such as insect and vertebrate OBP, and the NPC2, CSP, and CheA/B), and the presence of a transmembrane domain (characteristic of all chemosensory receptors, such as insect and vertebrate ORs, GRs and IRs). For that, we searched for a putative tissue-specific overrepresentation of such features in legs and palps (the candidate chemosensory structures in spiders) among the 3,353 transcripts with InterPro annotation. We found a significant over-representation of the signal peptide-encoding transcripts in legs-palps specific transcripts (Venn sections I, II and IV against the rest) (two tailed FET, *P*-value of 6.9 × 10^−3^), being especially evident for transcripts shared between palps and legs tissues (Venn section IV; two tailed FET, *P*-value of 9.7 × 10^−7^). Remarkably, the percentage of transcripts with signal peptide in section IV of the Venn diagram (transcripts expressed in both legs and palps, but not in ovary) is 27.8% (Fig. 5A), while the 40.6% of leg-specific transcripts have at least one transmembrane domain (Fig. 5B). Given that these features are not completely exclusive of chemosensory genes it is difficult to clearly assess whether these differences may reflect true differences in the chemosensory role of these tissues (see also Fig. S5).

The specific blast searches for chemosensory genes against the CheDB database detected several candidate transcripts. Nevertheless, the examination of the conceptual translation of these transcripts using HMM profiles showed that only seven candidates (two IR and five NPC2; Table S3) have the specific molecular signature of a chemosensory protein domain. Almost all the other candidates either exhibit non-chemosensory domain signatures or yielded no significant results in the search against HMM profiles. The two putative IR transcripts are specifically expressed in palps and each of them encodes a different Pfam domain characteristic of these receptors (Croset et al., 2010), the extracellular amino-terminal domain (PF01094; transcript Mcal_4794) and the ligand-gated ion channel domain (PF00060; transcript Mcal_5646). The closest related proteins of the *M. calpeiana* transcripts in the CheDB database correspond with two *S. mimosarum* predicted proteins annotated as “Glutamate receptor, ionotropic kainate 2” products (GenBank accessions KFM81344 and KFM59881, 48% and 67% of identity, with Mcal_4794 and Mcal_5646, respectively). Nevertheless, we cannot rule out that the two *M. calpeiana* transcripts were in fact two fragments of the same iGluR gene since KFM59881 is also a partial product that only includes the “Lig_chan” domain. Besides, the rest of best-hits in blast searches using these two *M. calpeiana* transcripts as queries correspond to kainate (KA) receptors followed by *α*-amino-3-hydroxy-5-methyl-4-isoxazole propionate (AMPA) members in other arthropod species. The phylogenetic trees of the members of these subfamilies in arthropods (built separately for each protein domain; see ‘Methods’) show that the translated proteins of Mcal_4794 and Mcal_5646 group in the same clade with some KA receptors of insects, centipedes or ticks (Figs. S6A and S6B), again suggesting their putative role in synaptic transmission and regulation (i.e., it would not be a chemosensory receptor).

The products of three of the five putative NPC2 encoding transcripts constitute a *M. calpeiana* specific monophyletic clade in the NPC2 family tree (Fig. S6C) and are specifically expressed in ovary, which is suggestive of a non-chemosensory function. The other two NPC2 are expressed in palp and legs (Mcal_1484) or palp-specific (Mcal_6333). Both encoding proteins are relatively distant to the *Apis mellifera* and *Camponotus japonicus* antennal expressed NPC2, being more related to some *I. scapularis* and *S. maritima* members as well as with the ovarian clade of NPC2. In light of these results, the possible chemosensory function of these proteins in palps and legs remains to be elucidated. These results strongly encourage further functional analyses to determine the putative chemosensory role of these NPC2 genes specifically expressed in palps and legs.

Recent genome sequencing projects have revealed that chelicerate genomes contain numerous copies of ionotropic (IR) and insect-like gustatory (GR) receptors, which are the principal candidates to perform chemoreceptor functions in these species. The apparent absence of genes belonging to these families specifically expressed in *M. calpeiana* palp/leg tissues might be explained by low sequence coverage. Many of these receptors are probably encoded by low expressed genes, and their detection might need more extensive sequencing. However, to date, there is no other study of the specific expression of either these receptors or other chemosensory family members in different tissues of a chelicerate. Given the life-style of *M. calpeiana*, i.e., it builds funnel-shaped webs, which it uses to trap prey, we cannot rule out the possibility of a residual role of a chemoreceptor system in favour of mechanoreception in this species. New deep sequencing transcriptomic data from other spider species are needed to answer this question. In fact, our preliminary results from tissue specific transcriptomes in *Dysdera silvatica* (Araneae, Haplogynae) (J Vizueta et al., 2015, unpublished data) indicate that members IRs and GRs families are specifically expressed in leg and palp tissues, suggesting their putative role in chemoreception in nocturnal running hunter spiders.

### Mygalomorph phylogeny

**Figure 6:**
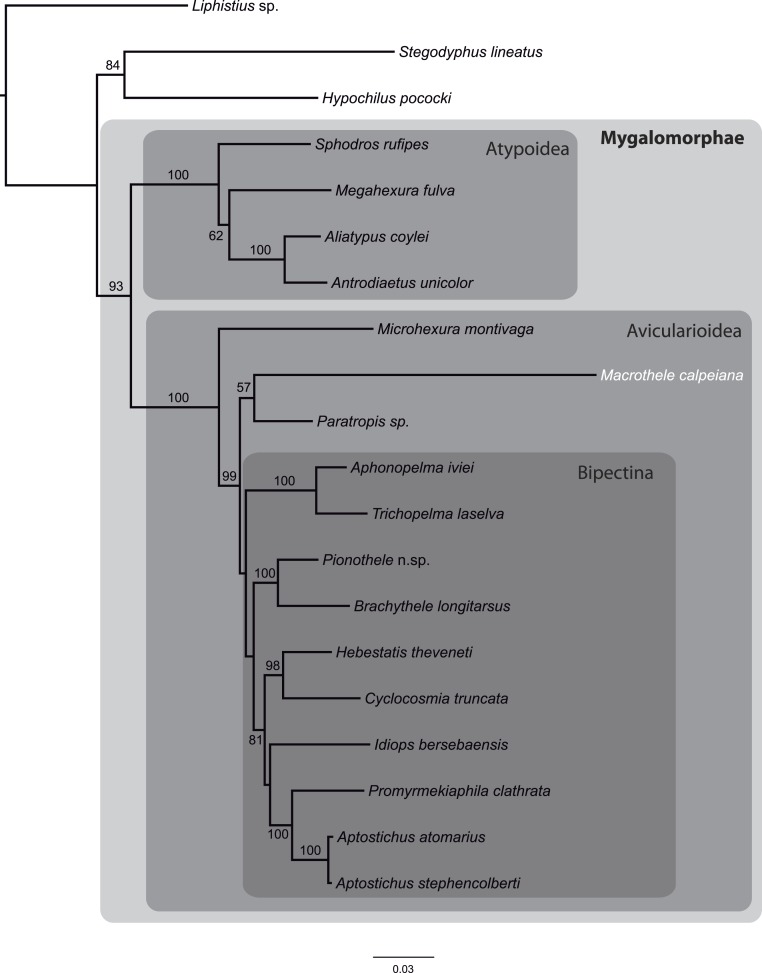
Phylogenetic relationships of major Mygalomorphae lineages sampled. ML tree showing the phylogenetic relationships of major Mygalomorphae lineages sampled. The analysis is based on a supermatrix of 35 putative orthologs (4,531 amino acids). Numbers indicate bootstrap support values >50%.

From the data matrix d327 of Bond et al. (2014), we built a new MSA with information of *M. calpeiana* obtained from our transcriptome analysis. We have filtered the data in order to include high quality homologous data with high coverage per taxon. Our final MSA comprises 17 Mygalomorph species (including *M. calpeiana*) and 3 non-mygalomorph outgroups (20 taxa; 35 genes; 4,531 amino acids; Table S8), with an average taxa coverage of 17.1. Our ML phylogenetic tree, rooted using *Liphistus* as an outgroup, mirrors those reported in Bond et al. (2014) and shows *M. calpeiana* as the sister lineage of the genus *Paratropis* (Fig. 6), albeit with low node support (57%), as part of the non-Bipectina Avicularioidea. Interestingly, in a recent study focused on the phylogenetic relationship and biogeographic origins of the genus *Macrothele* (Opatova & Arnedo, 2014) based on a denser taxonomic sampling but lower gene coverage (3 genes), a similar position of *Macrothele*, within the Aviculariodea but outside the Bipectina lineage, was also recovered.

## Conclusions

The tissue specific transcriptome presented here provides a novel resource for *Macrothele* researchers, and for people interested in spider systematics and molecular biology. Having ovary and non-ovary expressed transcripts-based markers, which may potentially differ in their evolutionary rates, can become instrumental for further studies aiming to understand the evolutionary processes acting at different time-scales, such as biological invasions, secondary gene flow or speciation, and to implement successful conservation polices; in particular, we have demonstrated the utility of these newly generated data by inferring the phylogenetic position of *M. calpeiana* in the Mygalomorphae tree. Moreover, our tissue-specific gene expression study represents a starting point to understanding the chemosensory system in spiders and, in general, in chelicerates.

## Supplemental Information

10.7717/peerj.1064/supp-1 Table S1 HK and CEG genes (A) List of the 5,752 *Drosophila melanogaster* and 3,786 human housekeeping genes used this study. (B) List of the Interpro domain associated with the chemosensory function.

10.7717/peerj.1064/supp-2 Table S2 DNA sequence of *Macrothele* transcripts DNA sequence of the 6,696 transcripts identified in *M. calpeiana*.

10.7717/peerj.1064/supp-3 Table S3 Functional annotation of the *M. calpeiana* transcripts Summary of the functional annotation of the *M. calpeiana* transcripts.

10.7717/peerj.1064/supp-4 Table S4 CEG genes identified in *Macrothele* CEG homologous genes identified in *M. calpeiana* transcripts.

10.7717/peerj.1064/supp-5 Table S5 *Macrothele* transcripts with significant HK or CEG blast hits *M. calpeiana* transcripts with significant HK or CEG blast hits

10.7717/peerj.1064/supp-6 Table S6 Gene Ontology terms over- and under-represented among *M. calpeiana* tissues List of the GO terms over- and under-represented among *M. calpeiana* tissues. (B) Analysis conducted excluding HK and CEG encoding genes (1,523 transcripts with GO annotation over 5,390 transcripts). (B) Analysis conducted using all data (2,619 transcripts with GO annotation over 6,619 transcripts).

10.7717/peerj.1064/supp-7 Table S7 KEGG pathways identified in *Macrothele* List of KEGG pathways identified in *M. calpeiana* transcripts.

10.7717/peerj.1064/supp-8 Table S8 Genes used in the molecular phylogenetics analyses List of the 35 genes used to infer the phylogenetic relationships among of major Mygalomorphae lineages sampled.

10.7717/peerj.1064/supp-9 Supplemental Information 9 Supplementary Figures

## Additional Information and Declarations

### Field Study Permissions

The following information was supplied relating to field study approvals (i.e., approving body and any reference numbers): Field permission from the Junta de Andalucia (Spain); reference: SGYB-AFR-CMM.

### DNA Deposition

The following information was supplied regarding the deposition of DNA sequences: http://www.ncbi.nlm.nih.gov/bioproject/PRJNA285862.
