# Supplementary material for: Comparative analysis of tissue-specific transcriptomes in the funnel-web spider Macrothele calpeiana (Araneae, Hexathelidae)
Source: PeerJ. 2015 Jun 30;3:e1064. doi: 10.7717/peerj.1064 (PMC4493671; doi:10.7717/peerj.1064)
Supplement: Supplemental Information 9 [file peerj-03-1064-s009.pdf]

Comparative analysis of tissue-specific transcriptomes in the funnel web spider *Macrothele calpeiana* (Araneae, Hexathelidae)

Cristina Frías-López, Francisca C. Almeida, Sara Guirao-Rico, Joel Vizueta, Alejandro Sánchez-Gracia, Miquel A. Arnedo and Julio Rozas

Supplementary Figures

Figure S1.

Distribution of the Gene Ontology (GO) terms associated with the complete set of *M. calpeiana* transcripts excluding singletons (transcripts formed by a single read). Panels A (for MF, molecular function) and B (for BP, Biological process) include information from the 1,734 transcripts with GO annotations over 3,467 sequences. Panels C (for MF, molecular function) and D (for BP, Biological process) include information from the 973 transcripts with GO annotations over 2,573 sequences (i.e. the 3,467 sequences after excluding HK or CEG genes).

Figure S2.

Number of *Macrothele* transcripts encoding HK or CEG genes.

Figure S3.

Venn diagrams showing the number of sequences excluding singletons expressed specifically in each tissue or in their intersections (blue, ochre and yellow indicate leg, palp and ovary, respectively). A) All transcripts (n = 3,467); B) Number of transcripts excluding those coding for housekeeping and CEG genes (n = 2,589); C) Number and percentage of transcripts coding for housekeeping genes (n = 688). D) Number and percentage of transcripts including in the CEG database (n = 533). In panels A and B, The area of each Venn diagram section is

approximately proportional to the number of transcripts (panels A and B), or to the particular fraction value (panels C and D).

Figure S4.

Differential distribution of the GO terms of the transcripts from leg or palp (Venn sections I, II and IV; in blue) and ovary (sections III, V, VI and VII; in red). Analysis comprising all transcripts with GO terms (2,619 transcripts over 6,696).

Figure S5.

Venn diagrams showing the percentage of specific interpro domains across tissues (the different Venn sections are indicated in roman numbers). Analysis conducted including HK and CEG encoding genes (3,353 transcripts with Interpro annotation over 6,696). A) Signal peptide domain. B) Transmembrane domain.

Figure S6

Phylogenetic relationships of a representative subset of arthropod iGluR (including IR8a/IR25a) and NPC2 family members. A) iGluR family tree based on “ANF_receptor” domain. Since we fail to detect the highly divergent “ANF_receptor” domain of IR8a and IR25a proteins in our HMM-search using the PF01094 profile (but see Croset et al., 2010) we did not include these sequences in the tree of this domain. B) iGluR family tree based on “Lig_chan” domain. C) NPC2 family tree, using the same protein identifiers as in Pelosi et al. (2014). NPC2 proteins expressed in the antennae of *A. melifera* and *C. japonicus* are indicated with an asterisk. Hexapods (*A. melifera*, *C. japonicum* and *D. melanogaster*), myriapods (*S. maritima*) and chelicerates (*I. scapularis*) sequences are shown in green, red and blue, respectively, while *M. calpeiana* are represented in shaded boxes. Numbers indicate node support values (percentage over 500 bootstrap replicates).

Figure S1A

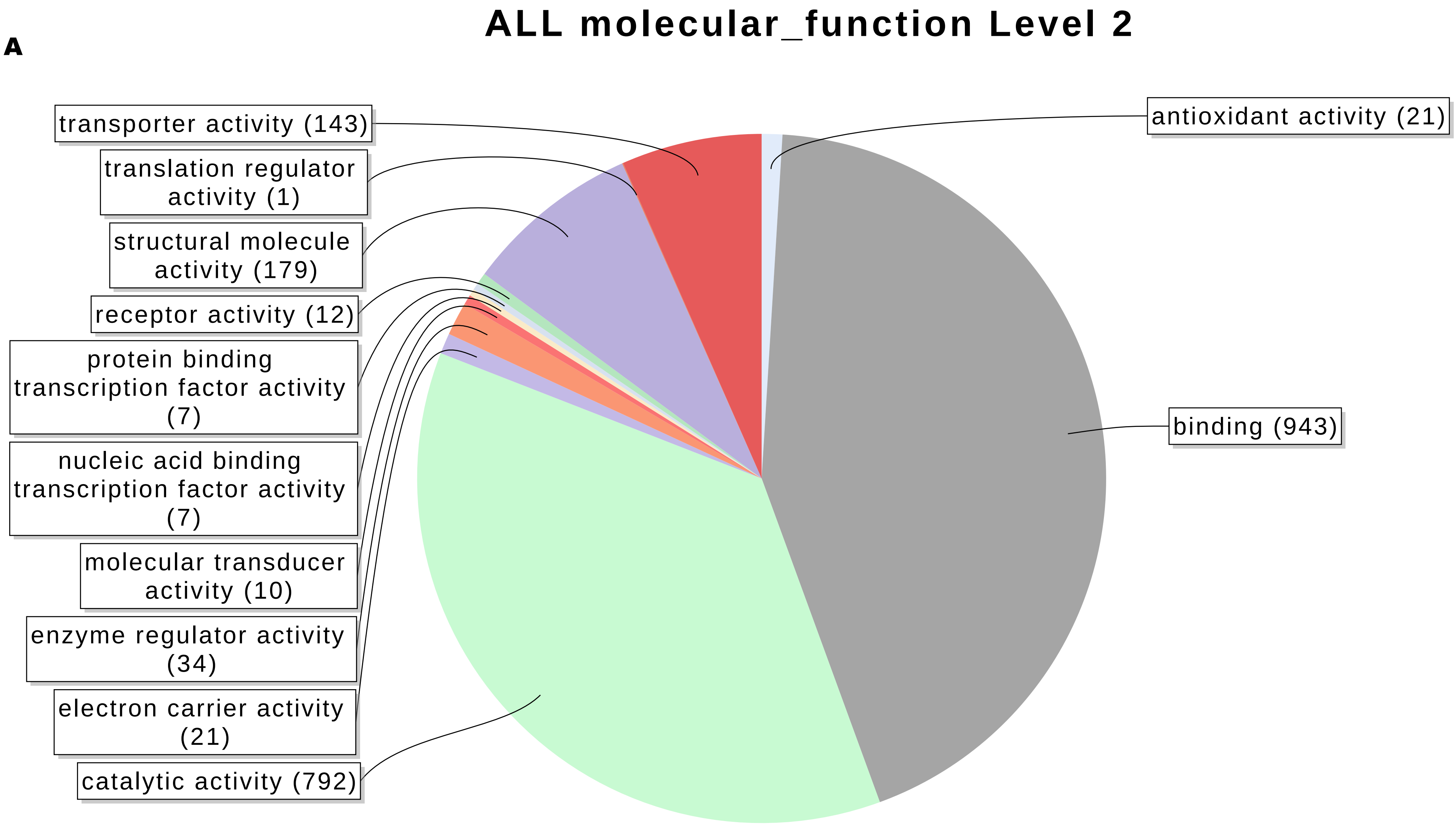

ALL molecular_function Level 2
transporter activity (143)
translation regulator activity (1)
structural molecule activity (179)
receptor activity (12)
protein binding transcription factor activity (7)
nucleic acid binding transcription factor activity (7)
molecular transducer activity (10)
enzyme regulator activity (34)
electron carrier activity (21)
catalytic activity (792)
antioxidant activity (21)
binding (943)

Figure S1B

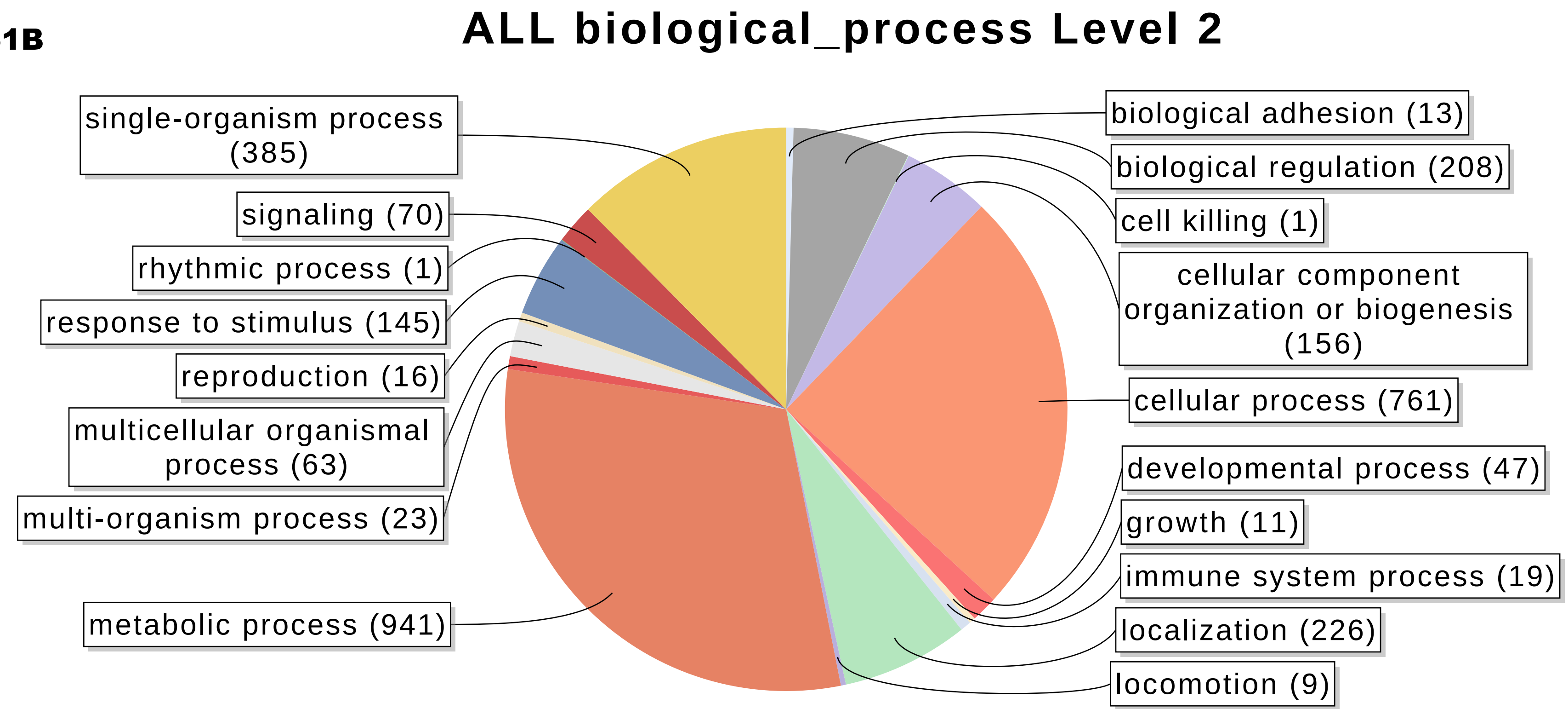
ALL biological_process Level 2
single-organism process (385)
signaling (70)
rhythmic process (1)
response to stimulus (145)
reproduction (16)
multicellular organismal process (63)
multi-organism process (23)
metabolic process (941)
biological adhesion (13)
biological regulation (208)
cell killing (1)
cellular component organization or biogenesis (156)
cellular process (761)
developmental process (47)
growth (11)
immune system process (19)
localization (226)
locomotion (9)

Figure S1C

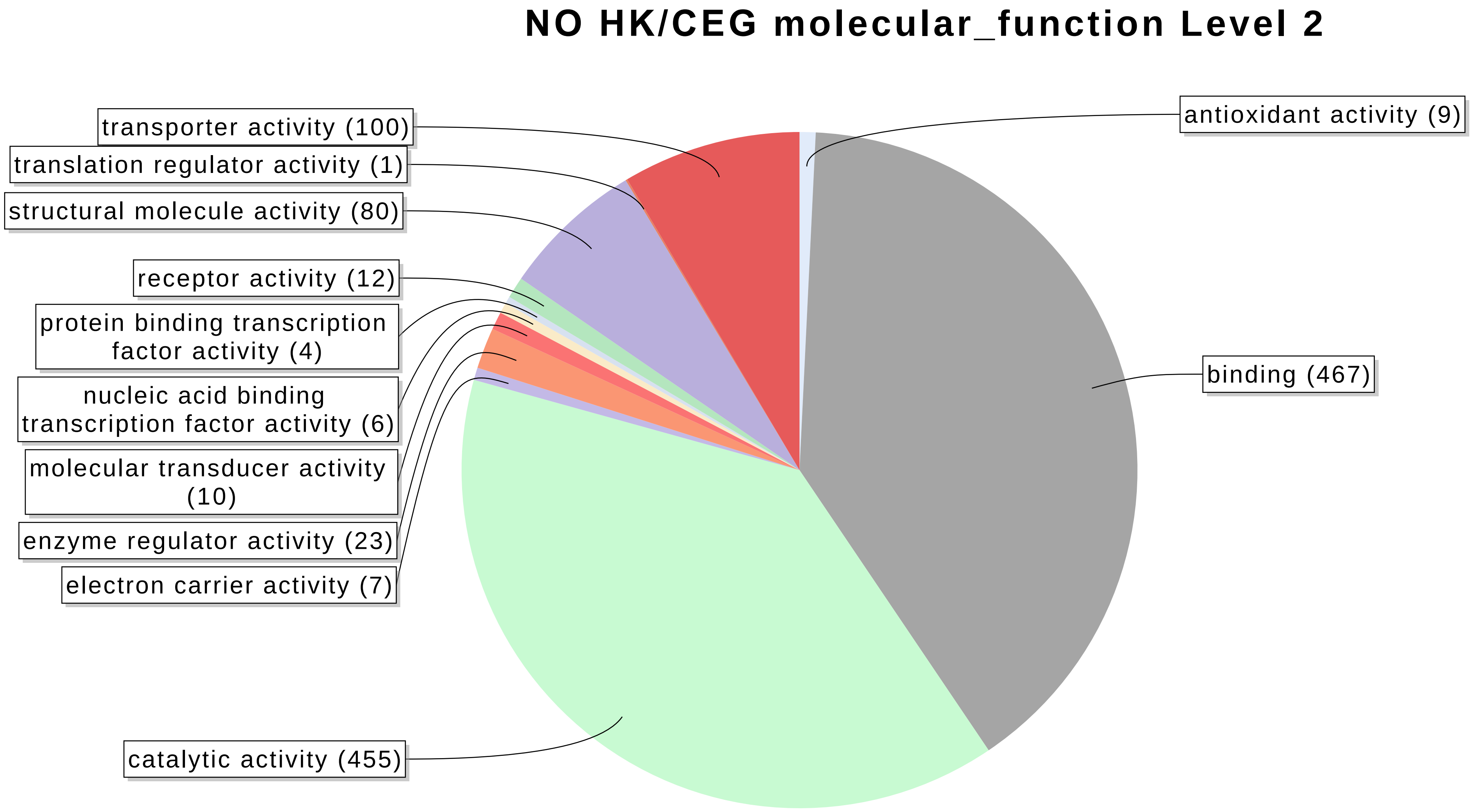
NO HK/CEG molecular_function Level 2
transporter activity (100)
translation regulator activity (1)
structural molecule activity (80)
receptor activity (12)
protein binding transcription factor activity (4)
nucleic acid binding transcription factor activity (6)
molecular transducer activity (10)
enzyme regulator activity (23)
electron carrier activity (7)
catalytic activity (455)
antioxidant activity (9)
binding (467)

Figure S1D

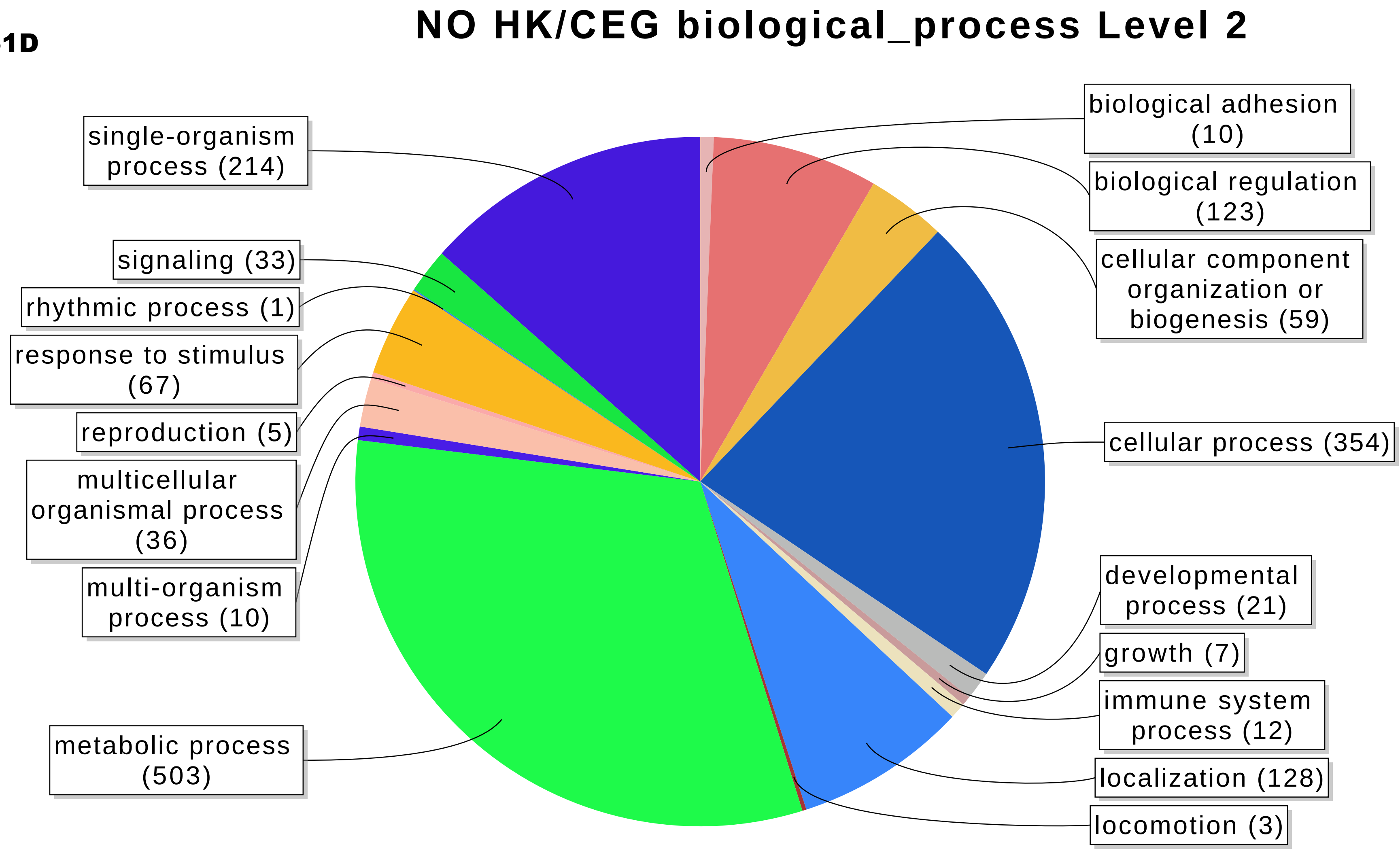

NO HK/CEG biological_process Level 2
single-organism process (214)
signaling (33)
rhythmic process (1)
response to stimulus (67)
reproduction (5)
multicellular organismal process (36)
multi-organism process (10)
metabolic process (503)
biological adhesion (10)
biological regulation (123)
cellular component organization or biogenesis (59)
cellular process (354)
developmental process (21)
growth (7)
immune system process (12)
localization (128)
locomotion (3)

Figure S2

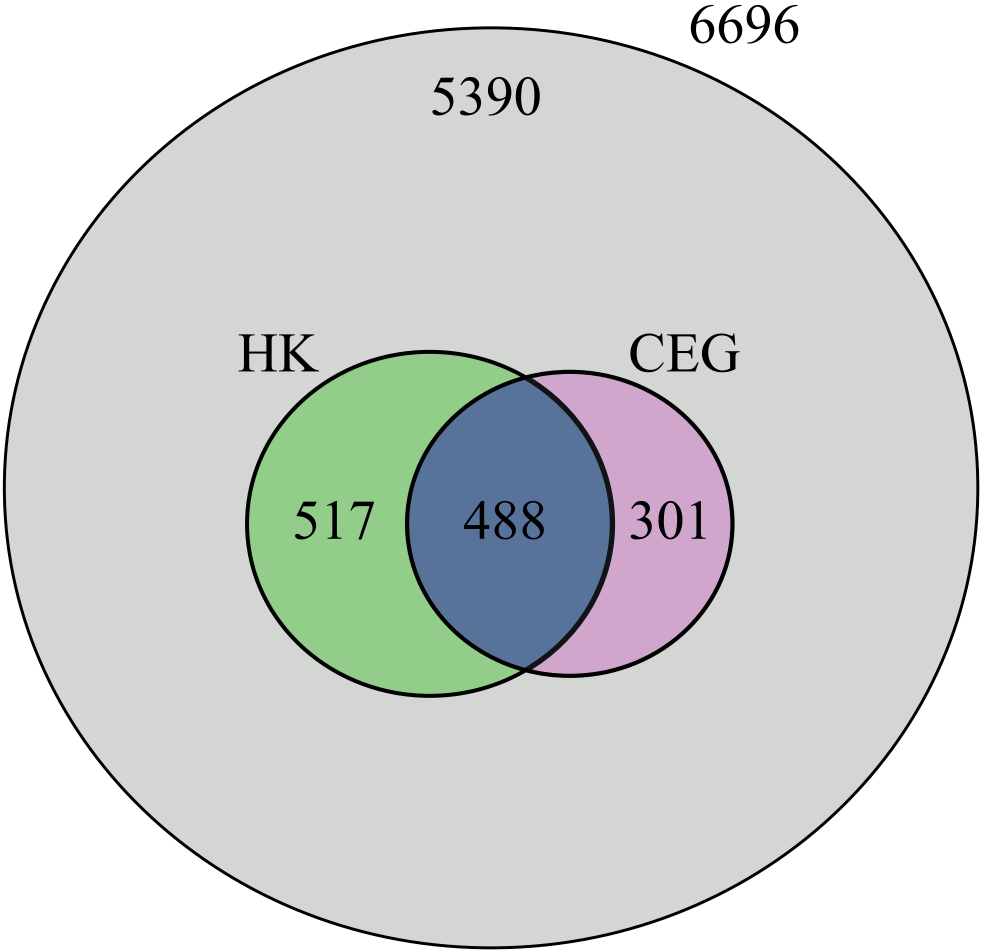

Figure S3

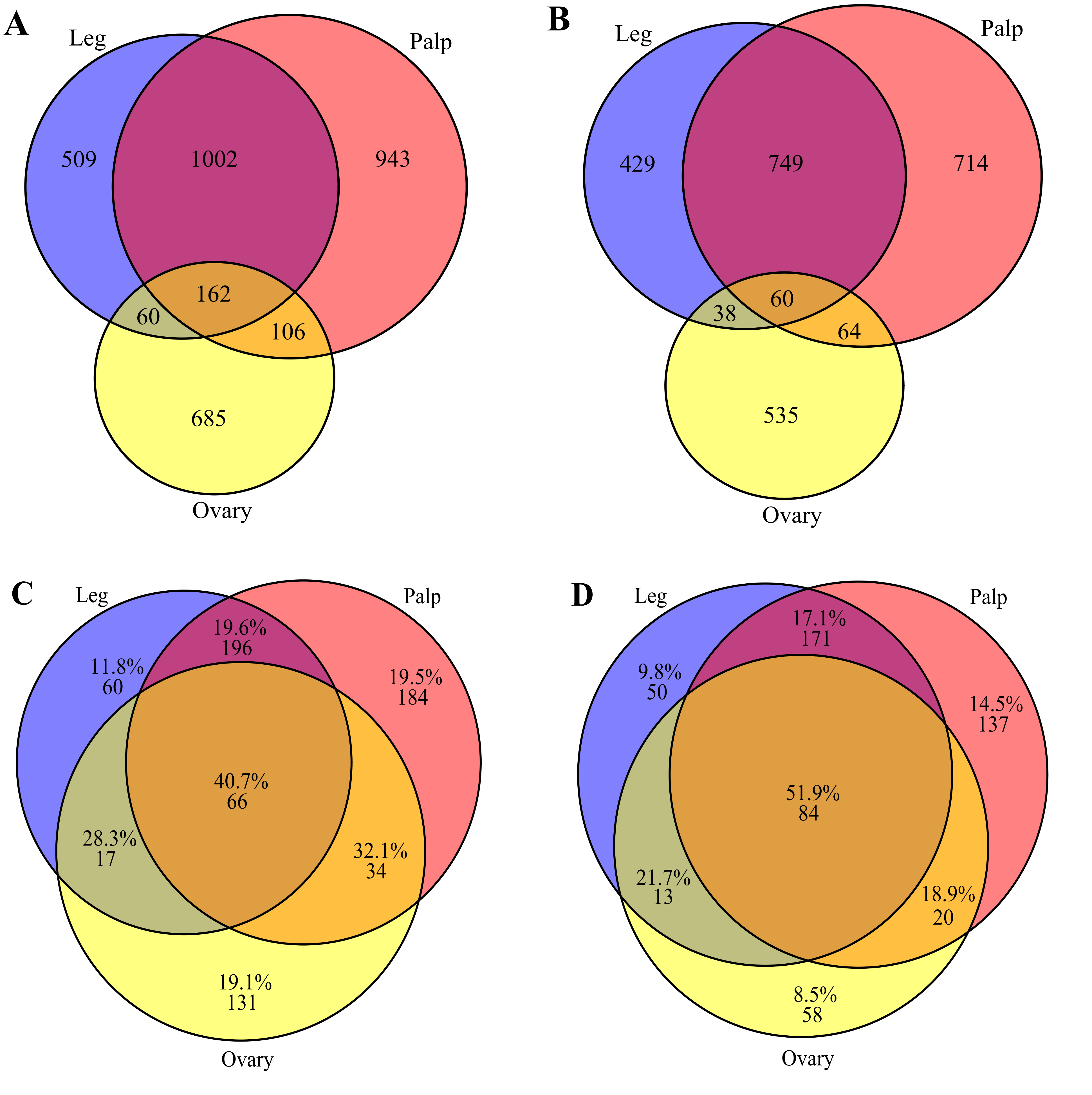
A
Leg
Palp
509
1002
943
162
60
106
685
Ovary
B
Leg
Palp
429
749
714
60
38
64
535
Ovary
C
Leg
Palp
19.6%
196
11.8%
60
19.5%
184
40.7%
66
28.3%
17
32.1%
34
19.1%
131
Ovary
D
Leg
Palp
17.1%
171
9.8%
50
14.5%
137
51.9%
84
21.7%
13
18.9%
20
8.5%
58
Ovary

Figure S4

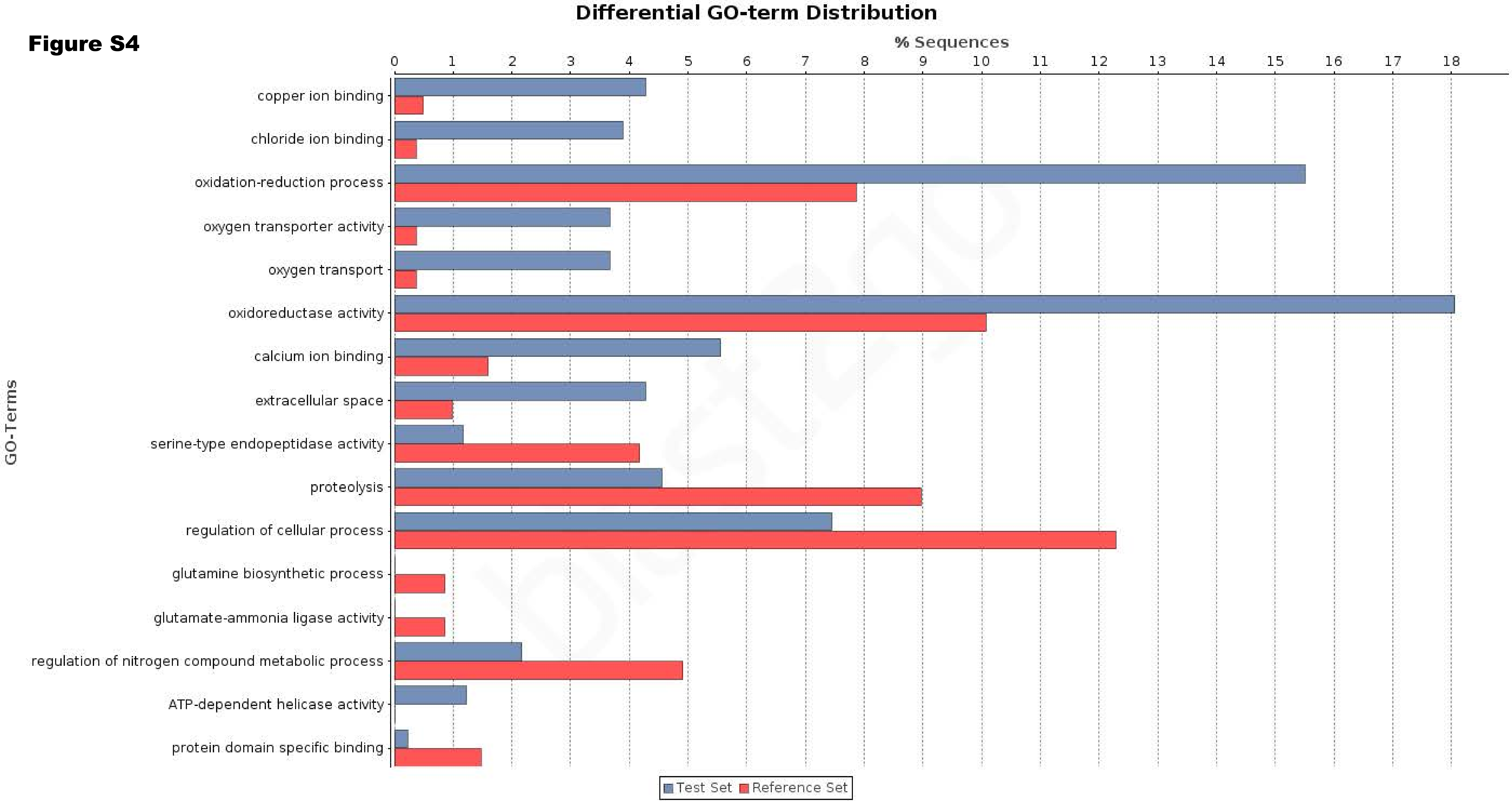
Differential GO-term Distribution
% Sequences
0
1
2
3
4
5
6
7
8
9
10
11
12
13
14
15
16
17
18
GO-Terms
copper ion binding
chloride ion binding
oxidation-reduction process
oxygen transporter activity
oxygen transport
oxidoreductase activity
calcium ion binding
extracellular space
serine-type endopeptidase activity
proteolysis
regulation of cellular process
glutamine biosynthetic process
glutamate-ammonia ligase activity
regulation of nitrogen compound metabolic process
ATP-dependent helicase activity
protein domain specific binding
Test Set
Reference Set

Figure S5A

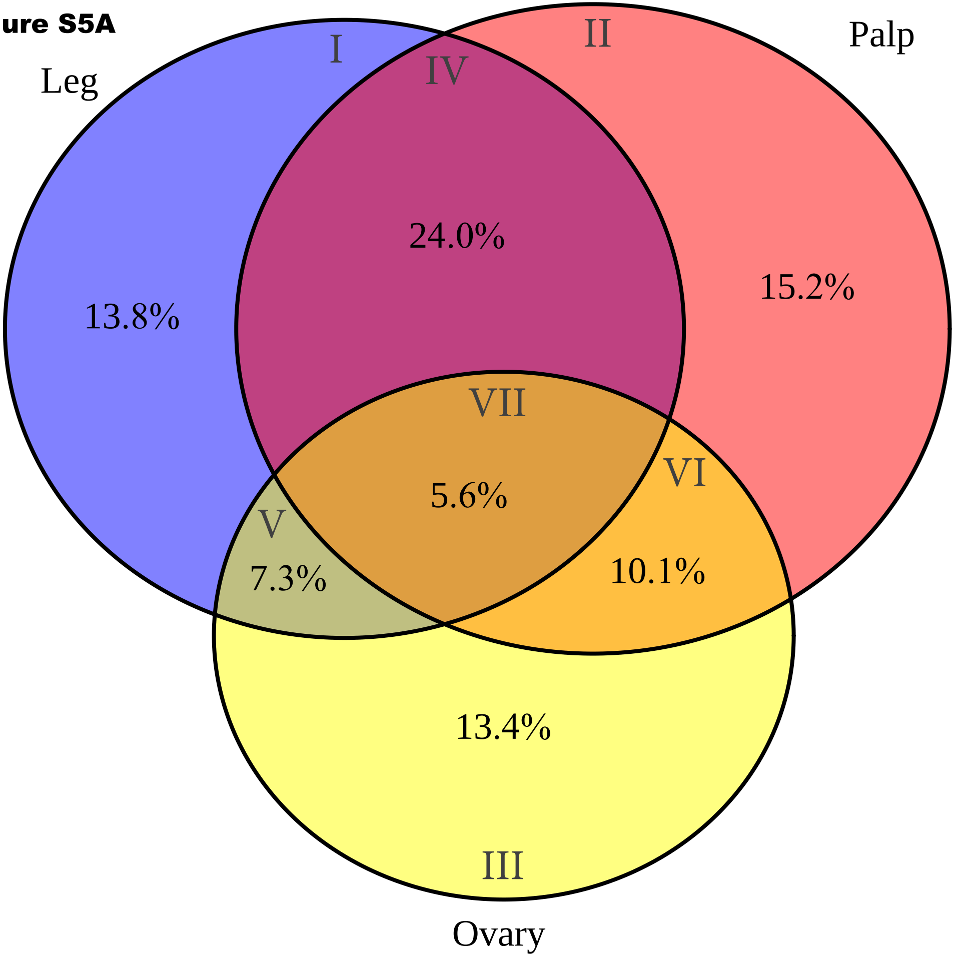

Figure S5B

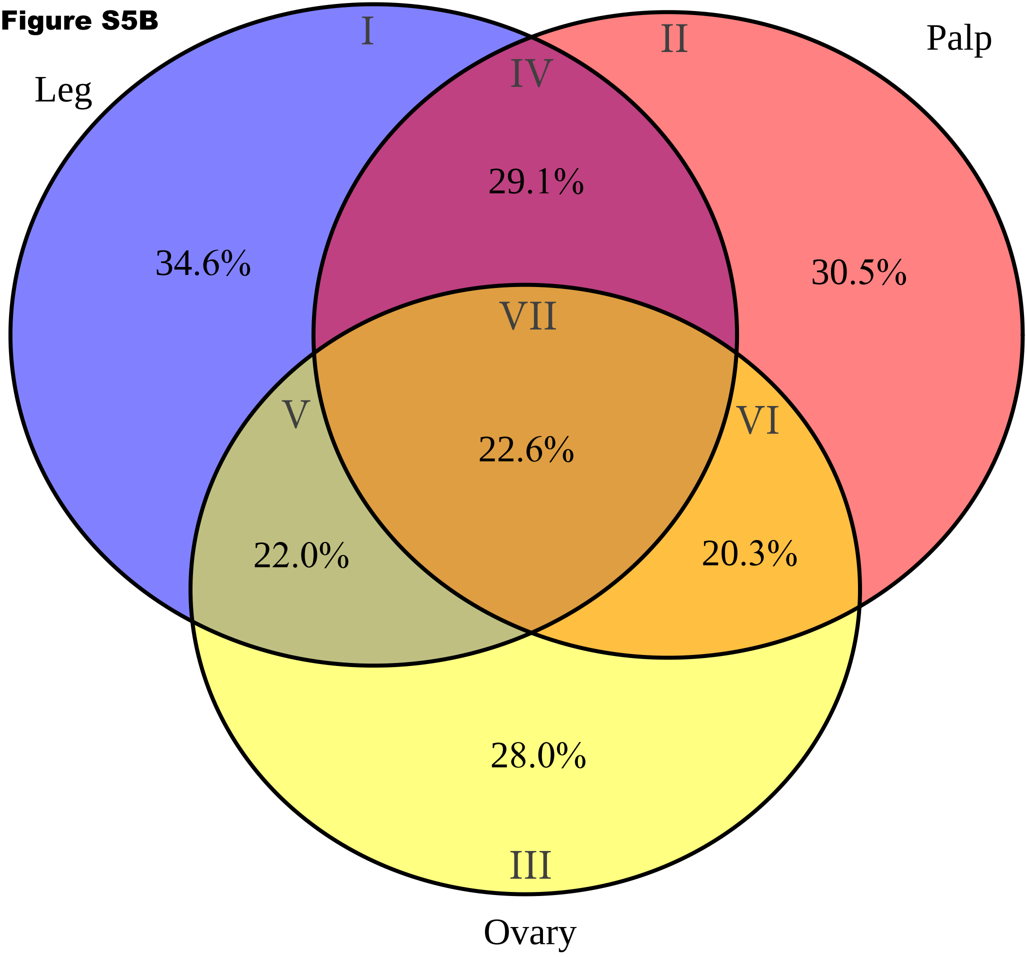

Figure 6A

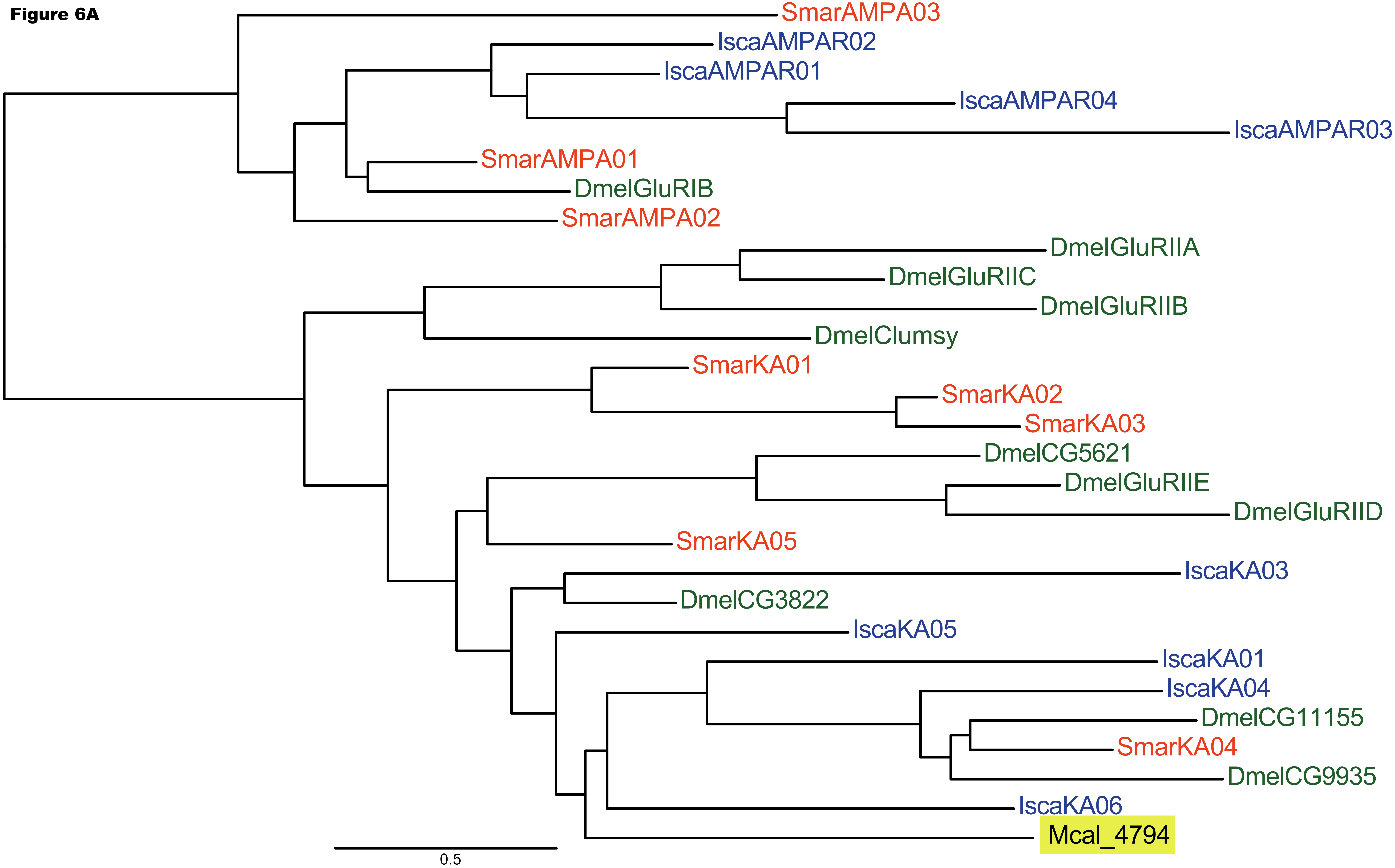
SmarAMPA03
IscaAMPAR02
IscaAMPAR01
IscaAMPAR04
IscaAMPAR03
SmarAMPA01
DmelGluRIB
SmarAMPA02
DmelGluRIIA
DmelGluRIIC
DmelGluRIIB
DmelClumsy
SmarKA01
SmarKA02
SmarKA03
DmelCG5621
DmelGluRIIE
DmelGluRIID
SmarKA05
IscaKA03
DmelCG3822
IscaKA05
IscaKA01
IscaKA04
DmelCG11155
SmarKA04
DmelCG9935
IscaKA06
Mcal_4794
0.5

Figure 6B

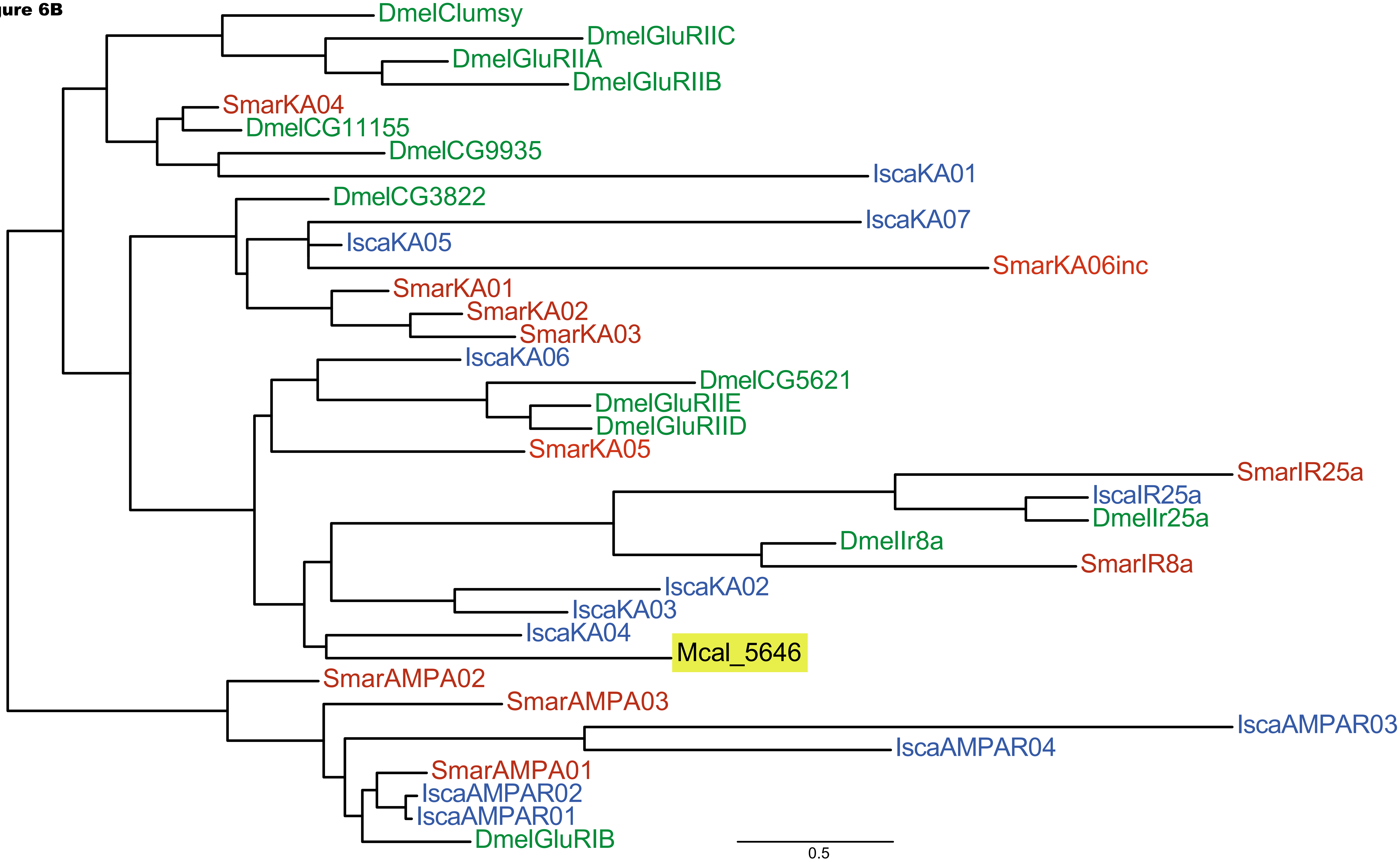
DmelClumsy
DmelGluRIIC
DmelGluRIIA
DmelGluRIIB
SmarKA04
DmelCG11155
DmelCG9935
IscaKA01
DmelCG3822
IscaKA07
IscaKA05
SmarKA06inc
SmarKA01
SmarKA02
SmarKA03
IscaKA06
DmelCG5621
DmelGluRIIE
DmelGluRIID
SmarKA05
SmarIR25a
IscaIR25a
DmelIr25a
DmelIr8a
SmarIR8a
IscaKA02
IscaKA03
IscaKA04
Mcal_5646
SmarAMPA02
SmarAMPA03
IscaAMPAR03
IscaAMPAR04
SmarAMPA01
IscaAMPAR02
IscaAMPAR01
DmelGluRIB
0.5

Figure 6C

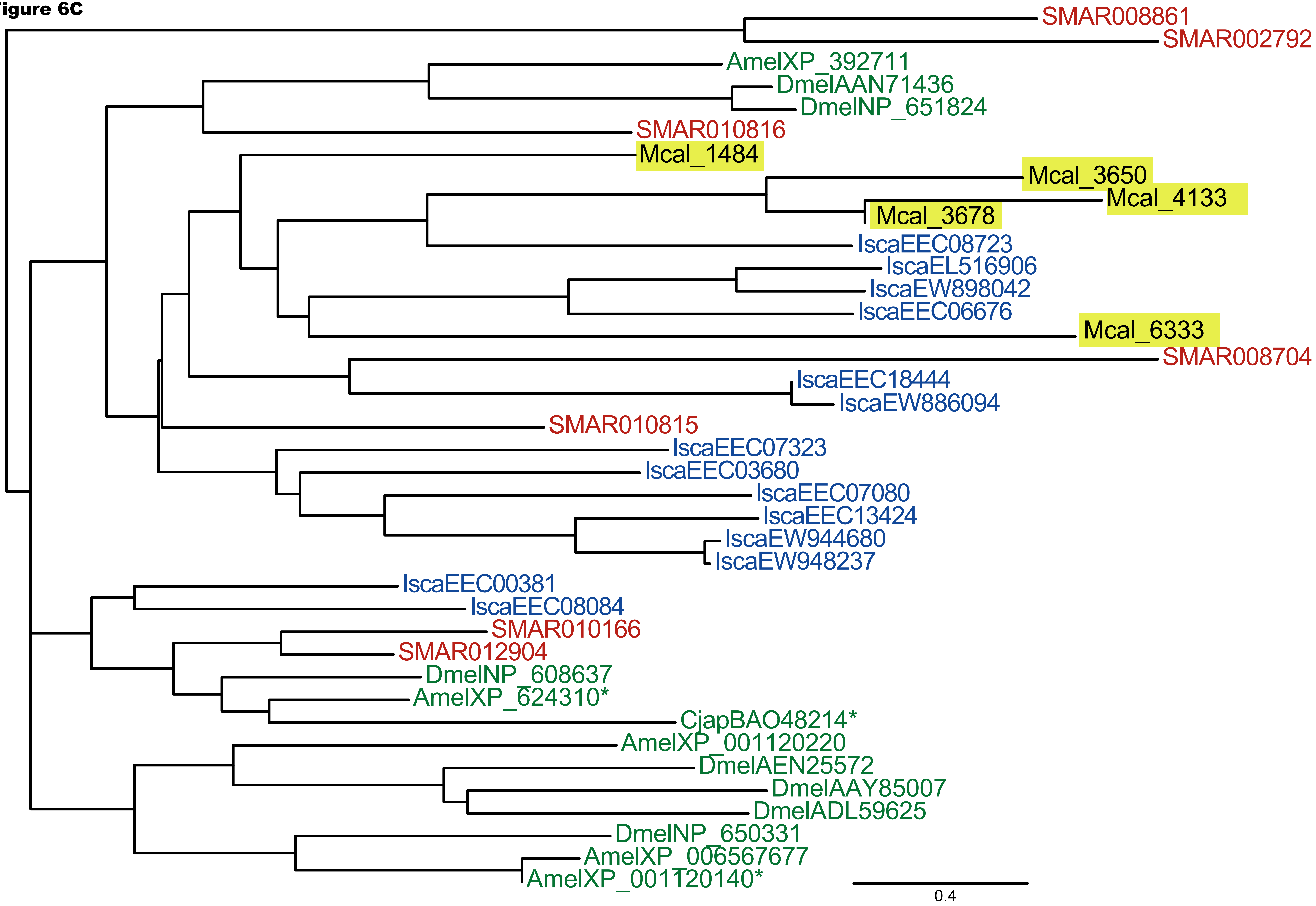

SMAR008861
SMAR002792
AmelXP_392711
DmelAAN71436
DmelNP_651824
SMAR010816
Mcal_1484
Mcal_3650
Mcal_4133
Mcal_3678
IscaEEC08723
IscaEL516906
IscaEW898042
IscaEEC06676
Mcal_6333
SMAR008704
IscaEEC18444
IscaEW886094
SMAR010815
IscaEEC07323
IscaEEC03680
IscaEEC07080
IscaEEC13424
IscaEW944680
IscaEW948237
IscaEEC00381
IscaEEC08084
SMAR010166
SMAR012904
DmelNP_608637
AmelXP_624310*
CjapBAO48214*
AmelXP_001120220
DmelAEN25572
DmelAAY85007
DmelADL59625
DmelNP_650331
AmelXP_006567677
AmelXP_001120140*
0.4
